# A Cross-Sectional Survey to Identify Current Pneumococcal Vaccination Practices and Barriers in Rural Community Pharmacies

**DOI:** 10.3390/vaccines13070756

**Published:** 2025-07-16

**Authors:** Ashley H. Chinchilla, Tyler C. Melton, Salisa C. Westrick, Tessa J. Hastings, Leticia Vieira, Grace T. Marley, Delesha M. Carpenter

**Affiliations:** 1 College of Pharmacy, University of Georgia, Athens, GA 30602, USA; 2 College of Pharmacy, Knoxville Campus, University of Tennessee Health Science Center, Knoxville, TN 37920, USA; tmelto11@uthsc.edu; 3 Harrison College of Pharmacy, Auburn University, Auburn, AL 36849, USA; westrsc@auburn.edu; 4 College of Pharmacy, University of South Carolina, Columbia, SC 29208, USA; hastint@mailbox.sc.edu; 5 Eshelman School of Pharmacy, University of North Carolina at Chapel Hill, Chapel Hill, NC 27599, USA; vleticia@unc.edu (L.V.); grace_trull@unc.edu (G.T.M.); dmcarpenter@unc.edu (D.M.C.)

**Keywords:** pneumococcal vaccine, immunization barriers, community pharmacy, rural health

## Abstract

**Background**: Pneumococcal vaccination rates in the United States (US) remain suboptimal, especially for adults aged 19 to 64 with high-risk medical conditions. Community-pharmacy-based immunization services increase vaccine access, particularly in rural areas. This study describes the provision of pneumococcal immunization services, assesses the processes used to identify and confirm patient eligibility, and determines barriers to immunization services in rural community pharmacies. **Methods**: A cross-sectional survey was emailed to members of the Rural Research Alliance of Community Pharmacies, located in the southeastern US. The survey assessed which pneumococcal vaccines were offered, age groups, prescription requirements, and how patient eligibility was determined. In addition, participants were asked to rate a series of patient-related and organizational barriers to pneumococcal vaccination. **Results**: Ninety-four pharmacies completed the survey, with most (96.8%) offering pneumococcal vaccines, most commonly PCV20 (95.6%). Most pharmacies vaccinated patients upon request (98.9%) or when patients presented with a prescription (82.4%), but few proactively contacted patients to schedule the vaccination (17.6%). Pharmacists most often administered pneumococcal vaccines to patients aged 65 and older and used patient age and immunization information systems to identify eligible patients. The most common patient-related barrier was the patient’s belief that they do not need the vaccine. The most common organizational barriers were inadequate reimbursements for vaccine administration and vaccine products. **Conclusions**: Pneumococcal vaccinations are commonly offered in rural community pharmacies, which play an important role in immunization access. With recent guideline changes to the age-based recommendation, there is an opportunity to optimize strategies to increase vaccine uptake.

## 1. Introduction

Streptococcus pneumoniae is a leading cause of community-acquired pneumonia and bacterial meningitis in the United States (US) [1]. There are more than 150,000 hospitalizations from pneumococcal pneumonia every year, and an additional 2000 cases of pneumococcal meningitis and 5000 cases of bacteremia without pneumonia occur annually [1]. In addition, the treatment of pneumococcal disease has become more challenging as antibiotic resistance has become more prevalent. A recent study noted resistance to one or more antibiotics found in 37.7% of streptococcus pneumoniae cultures [2].

Vaccines for the prevention of pneumococcal disease have been available in the US for several years and have made a significant positive impact on disease burden [1]. However, vaccine uptake remains low in some adults. While 64% of adults aged 65 and older have received a pneumococcal vaccine, only 23% of adults aged 19–64 with one or more risk factors have been vaccinated against pneumococcal disease [3].

Several multi-level barriers to adult vaccination have been reported in the literature, including financial barriers for the patient (e.g., no insurance coverage), low perceived patient risk sometimes due to knowledge gaps about vaccination or the disease, a lack of provider recommendation, the cost associated with vaccine supply, and a lack of reimbursement for pharmacy administration [4,5,6,7]. While adult immunization rates for several vaccines are suboptimal, pneumococcal vaccination lags behind others, including tetanus and influenza [3]. In addition, immunization rates are lower for patients who live in rural communities [8,9]. This trend is not unique to the US, as patients in rural areas in several other countries also have lower vaccination rates compared to their urban counterparts [10,11].

Compared to other immunizations, pneumococcal vaccines have unique challenges, as there have been several changes to clinical recommendations in recent years. Since 2021, three new conjugate pneumococcal vaccines have been approved and incorporated into adult immunization recommendations by the Advisory Committee on Immunization Practices (ACIP). Most recently, the ACIP updated the age-based recommendation for pneumococcal vaccination, decreasing the recommended age from 65 and older to age 50 and older [12].

Immunization services are commonly offered in community-based pharmacies, and studies have demonstrated the positive impact of pharmacist involvement in immunization practices [4,13]. Nearly 90% of Americans live within five miles of a pharmacy, making pharmacists highly accessible healthcare providers [14]. In fact, the National Vaccine Advisory Committee has recommended collaborating with pharmacists as a strategy to increase vaccine access and decrease immunization inequity, particularly in rural communities [15].

Understanding the current landscape of pneumococcal vaccination services is important to improve immunization rates, especially given recent recommendation changes. This study aims to describe the provision of pneumococcal immunization services, assess the processes used to identify and confirm patient eligibility, and determine barriers to pneumococcal immunization services in rural community pharmacies.

## 2. Materials and Methods

### 2.1. Study Design and Setting

This study employed a cross-sectional design using an online Qualtrics survey of 153 pharmacists who are members of the Rural Research Alliance of Community Pharmacies (RURAL-CP). The RURAL-CP is a multi-state practice-based research network developed exclusively for rural community pharmacies, with members located in seven southeastern states (Alabama, Arkansas, Georgia, Mississippi, North Carolina, South Carolina, and Tennessee) [16]. To be eligible for participation, pharmacists must be licensed, speak English, and have been employed at a RURAL-CP pharmacy for at least one month. RURAL-CP pharmacies are located in rural areas, which are defined as either having a Rural–Urban Commuting Area (RUCA) code [17] greater than or equal to four or, in cases where the pharmacy’s RUCA score is inflated due to proximity to a major metropolitan area, having a pharmacy that is located in a town that has a population of fewer than 2500 people; is located in a county that has a population that is more than 50% rural; or is at least one hour from the nearest major city.

### 2.2. Recruitment and Data Collection

Recruitment took place from July to October 2024, during which time RURAL-CP pharmacists received an initial recruitment email and non-completers received up to four reminder emails. Those who completed the survey received a USD 75 incentive. Respondents were assigned study identification numbers so individual pharmacists could not be identified. The study protocol was reviewed by the University of North Carolina Institutional Review Board, which determined the protocol to be exempt (IRB #: 24-0538).

### 2.3. Measures

The survey took approximately 25 min to complete and included questions related to current pneumococcal vaccination services, processes used to identify and confirm eligible patients, and barriers to providing vaccines (full survey included as Supplementary Material). Due to a lack of existing validated pneumococcal measures, most survey questions were newly developed, except for the patient-related and organizational barriers, which were adapted from Westrick and colleagues (2018). New survey questions were reviewed for face validity and revised by the investigator team, which consisted of pharmacists and health services researchers.

Participants answered several questions about their pharmacy’s provision of pneumococcal immunization services, such as whether the respondent’s pharmacy offered pneumococcal vaccines in 2024, the types and doses of pneumococcal vaccines administered in 2023, preferred regimens for different patient groups, the age groups of pneumococcal vaccine recipients, and whether a prescription was required. Next, participants were asked about the processes they used to identify and confirm the eligibility of patients.

The survey also assessed 11 patient-related and 17 organizational barriers to pneumococcal immunization service provision. Examples of patient barriers included patients “refusing vaccine because they do not think they need it” and “being too busy to discuss the vaccine with the pharmacist,” while organizational barriers included lack of time to prepare and administer the vaccine and inadequate reimbursement. Response options ranged from 1 (not at all/not applicable) to 5 (an extreme barrier).

Participants also answered 8 demographic questions, including age, race, experience, type of pharmacy, and geographic location.

All data were imported into IBM SPSS Statistics version 30 (IBM Corp., Armonk, NY, USA) for analysis. Descriptive statistics were calculated to characterize the sample and describe the pharmacy’s provision of pneumococcal immunization services, the processes to identify and confirm the eligibility of patients, and patient and organizational barriers.

## 3. Results

Ninety-four pharmacists, representing 94 different pharmacies, completed the survey for a response rate of 61.4%. The overwhelming majority of pharmacies offered pneumococcal vaccines (96.8%) with only three not offering the vaccine. Reasons for not offering the pneumococcal vaccine included a lack of resources, the need for staff training, having too much competition, and the cost associated with Medicare enrollment, a federal health insurance program for older adults. Table 1 shows the participants’ individual and pharmacy characteristics for the remaining responses. The participants were evenly split by gender (50% male; 50% female). The majority of participants were White or Caucasian (95.7%), held a PharmD degree (75.3%), had been working as pharmacists for 16 years or more (52.7%), and were pharmacy managers (54.3%). Further, the majority worked in a single independent pharmacy (59.6%), with an average age of 44 years.

In 2024, the most commonly offered pneumococcal vaccine was PCV20, provided by 95.6% of pharmacies, followed by PPSV23 (62.6%) and PCV15 (22.0%) (Table 2). In 2023, the average number of PCV20 doses administered was 34.1, with a range of 1 to 160 doses. A significant majority of pharmacies (91.1%) co-administered pneumococcal vaccines with other vaccines. For both risk-based and age-based recommendation groups, an overwhelming majority of participants preferred PCV20.

When asked about the frequency of pneumococcal vaccine administration, the results varied significantly across different patient age groups (Table 3). Most pharmacies never administered these vaccines to children aged 5 years and younger (97.8%), compared to 87.9% for those aged 5–18. The proportion of pharmacies administering the vaccine increased with the age of the patients: 92.3% of participants administered pneumococcal vaccines for those aged 19–64, and all participants administered the vaccine for those 65 and older. Regarding prescription requirements, the proportion of pharmacies requiring a prescription for the pneumococcal vaccine decreased as the age of the vaccine recipient increased.

Table 4 shows strategies and resources used to improve pneumococcal vaccine uptake, with the two most common strategies being vaccinating patients upon request (98.9%) and vaccinating individuals who had a prescription from their provider (82.4%). Proactively contacting patients to schedule vaccinations was the least commonly used strategy (17.6%). To identify eligible patients, participants primarily used patient age (74.7%) and Immunization Information Systems (71.4%), with medication refill history being less frequently used (39.6%). For confirming patient eligibility, Immunization Information Systems (91.2%) and patient age (86.8%) were the main data sources, followed by medication refill history (61.5%). The CDC immunization schedule was the primary resource for determining patient eligibility (80.2%), with other tools like the PneumoRecs Vax Advisor Website or Mobile App (37.4%) and previous training information (34.1%) also being utilized.

Overall, participants did not report that many patient-related or organizational barriers were majorly problematic (Table 5). The most prominent patient-related barrier identified was the patients’ perceived belief that they do not need the vaccine (Mean = 3.1, S.D. = 1.1). Regarding organizational barriers, the top concerns were inadequate reimbursements for vaccine administration (mean = 3.0, S.D. = 1.5) and vaccine products (mean = 3.1, S.D. = 1.5).

## 4. Discussion

This study explores the provision of pneumococcal immunization services, identifies processes used to recognize and confirm patient eligibility, and describes barriers to vaccination in rural community pharmacies. The findings highlight the vital role rural pharmacies play in expanding access to pneumococcal immunization services and can inform the development of targeted policies and interventions to improve vaccine uptake and ultimately save lives.

An important aspect of the study to consider is how pneumococcal vaccine preferences align with immunization recommendations. Nearly all surveyed pharmacies (96.8%) offered pneumococcal vaccines, demonstrating the accessibility of immunization services in rural pharmacies. The majority of pharmacists reported administering PCV20, reflecting widespread alignment with Advisory Committee on Immunization Practices (ACIP) guidelines that support this vaccine for its broader serotype coverage and simplified schedule [12]. PCV20’s popularity over PCV15 and PPSV23 suggests that rural pharmacists are not only aware of current recommendations but also integrating them effectively into routine practice. While this study was conducted before the release of PCV21, future investigations will be essential to evaluate the new vaccine’s uptake and effect on immunization workflows in community pharmacies.

Vaccination practices varied with patient age, which offers insights into where improvement in immunization services is possible. Specifically, pharmacists reported that pneumococcal vaccination was most frequently provided to adults aged 65 and older, with significantly lower rates reported for those between 19 and 64 years of age. While this trend reflects the ACIP guidelines in effect during the study period, it also highlights opportunities to promote targeted immunization strategies to include newly expanded recommendations for adults aged 50 and older. Given that pharmacists commonly rely on patient age and Immunization Information Systems to determine vaccine eligibility, adapting workflows to proactively identify and immunize this newly eligible group could lead to the expansion of evidence-based pneumococcal vaccine coverage in rural areas [18,19]. Notably, only 17.6% of pharmacists reported proactively contacting patients, identifying a key area to target to improve vaccine uptake. By examining age-based vaccination trends in rural areas, pharmacies can adapt existing vaccination processes to include proactive involvement in communicating vaccination eligibility to at-risk patients and creating targeted opportunities to encourage vaccine uptake.

Despite efforts to improve vaccination rates, several barriers hindered vaccine uptake, including perceived patient attitudes toward the vaccine and reimbursement challenges encountered by community pharmacies. The most frequently reported patient-related barrier to immunization was the belief that vaccination was unnecessary, which is consistent with findings from prior studies on vaccine hesitancy [20,21]. Patient attitudes toward vaccination in general have evolved in recent years both in the US and other countries, with increasing hesitancy toward vaccines [10,20]. Patient concerns can be addressed with targeted vaccine communication, where pharmacists discuss health risks concerning pneumococcal disease for patients who are qualified to receive the vaccine and the benefits of vaccination. Pharmacy barriers, such as inadequate reimbursement for vaccine administration and product acquisition, also emerged as concerns. In the US, provider reimbursement for vaccines and healthcare services can vary widely based on the payor. Patients may be covered by various health insurance plans, through either a private company or the government, that oftentimes require no patient cost sharing, or may be uninsured and pay the full cost of healthcare services. Pharmacies may see even more variability in reimbursement in the future with the expansion of the age-based recommendation. Inadequate reimbursement creates financial strain for community pharmacies, which negatively impacts pharmacy readiness and willingness to offer vaccine services [22]. While time-related barriers were generally considered minimal, pharmacists reported logistical challenges in tracking multiple dose regimens and a lack of systems for routine screening. Perhaps this limitation in technology could be corrected by pharmacies using a different dispensing and operation system. In summary, this study emphasizes the need for both organization-level and patient-education interventions to improve vaccine delivery.

When compared to other routinely administered vaccines, such as influenza, pneumococcal vaccines present unique challenges and opportunities for vaccine uptake, patient awareness, and delivery [21,23]. The use of technology and tools, such as vaccine scheduling software, is necessary to streamline providing immunization services [24]. While the CDC adult immunization schedule was widely used by the study participants, the PneumoRecs VaxAdvisor application was only used by 37% of participants to assess patient eligibility or during pneumococcal vaccine recommendation discussions. Despite limitations in technology being a barrier to providing pneumococcal vaccinations, pharmacists did not utilize free evidence-based applications offered by reputable sources such as the CDC. This creates missed opportunities to streamline eligibility assessments; however, it is unclear if this underutilization of technology is due to a gap in knowledge or limited availability. The improved adoption of digital tools may support more efficient and accurate vaccination decision-making [24].

Translating the insights of this study into practice and policy is essential for the development of sustainable improvements in community pharmacy immunization services. Several actionable insights have emerged from this study, including patient education, reimbursement opportunities, proactive communication, and workflow integration. Patient education is essential to assist in dispelling misinformation, improving understanding of vaccine benefits, and promoting vaccine uptake [25]. Enhancing financial incentives and modifying current reimbursement structures to include adequate compensation for vaccination services is essential for the continuation and participation of community pharmacists in providing vaccines. Proactive communication is also necessary to identify eligible patients, as is providing patient education and promoting the development of trusted pharmacist relations necessary to overcome vaccine hesitancy [25]. Lastly, the incorporation of mobile applications and decision-support tools into pharmacy practice can ease community pharmacy challenges, such as scheduling and vaccine dose tracking, while ensuring consistency with ACIP recommendations. These insights are relevant in rural settings, where pharmacies often serve as a primary access point, or in some cases, the only point, for preventive healthcare.

This study has several limitations. The study participants were predominantly rural independent pharmacies located in seven southeastern states, which may limit generalizability to urban environments, other states, and other pharmacy types. In addition, the surveys were self-reported, which may have introduced the potential for response bias. Future studies should consider broader geographic representation, the inclusion of chain pharmacies, and the use of pharmacy records. Additional research is also needed to assess the impact of the ACIP’s updated recommendations and the introduction of PCV21 on pneumococcal vaccination practices and patient outcomes.

## 5. Conclusions

Rural community pharmacies can play a crucial role in increasing adult pneumococcal immunization rates. Despite current success in vaccine accessibility and alignment with guidelines, opportunities remain to improve vaccine uptake through enhanced patient education, reimbursement reforms, and more proactive engagement strategies, especially considering the expanded age-based recommendations. As guidelines evolve and new vaccines become available, rural pharmacies will continue to play an essential role in addressing immunization accessibility and promoting public health.

## Figures and Tables

**Table 1:** Participant and pharmacy characteristics of those offering pneumococcal vaccines.

	*n* (%)
Gender (N = 91)	
Male	44 (48.4)
Female	47 (51.6)
Race (N = 90)	
White or Caucasian	86 (95.5)
Black or African American	2 (2.2)
Other	3 (3.3)
Highest education (N = 90)	
PharmD	68 (75.6)
BS Pharmacy	19 (21.1)
Other	3 (3.3)
Length of working as a pharmacist (N = 90)	
1–3 years	7 (7.8)
4–7 years	8 (8.9)
8–11 years	13 (14.4)
12–15 years	13 (14.4)
≥16 years	49 (54.4)
Position (N = 91)	
Pharmacy manager	50 (54.9)
Pharmacist	25 (27.4)
Other ^a^	16 (17.6)
Type of pharmacy (N = 91)	
A single independent pharmacy	54 (59.3)
An independent pharmacy with multiple locations	34 (37.4)
Grocery store chain	3 (3.3)
Geographic location of pharmacy (N = 91)	
Alabama	9 (9.9)
Arkansas	13 (14.3)
Georgia	15 (16.5)
Mississippi	7 (7.7)
North Carolina	16 (17.6)
South Carolina	15 (16.5)
Tennessee	16 (17.6)
	Mean (S.D.)
Age (N = 91)	44 (11.6)
	Range 26–77

^a^ Owner; operations manager.

**Table 2:** Provision of pneumococcal immunization services (N = 91).

	*n* (%)
Type of pneumococcal vaccines offered in 2024	
PCV15	20 (22.0)
PCV20	87 (95.6)
PPSV23	57 (62.6)
Number of pneumococcal vaccine doses administered in 2023	
PCV13 ^a^ (n = 28)	9.4 (12.5); range 1–50
PCV15 (n = 8)	3.9 (1.6); range 1–5
PCV20 (n = 88)	34.1 (38.4); range 1–160
PPSV23 (n = 57)	12.3 (16.9); range 1–100
Whether pharmacy co-administers pneumococcal vaccines with other vaccines (N = 91)	
Yes	83 (91.1)
Preferred pneumococcal regimen for adults, aged 19–64 with high-risk conditions	
PCV20	79 (86.8)
PCV15, followed by PPSV23	5 (5.5)
No preference	5 (5.5)
Preferred pneumococcal regimen for adults, aged ≥ 65 ^b^	
PCV20	80 (87.9)
PCV15, followed by PPSV23	3 (3.3)
No preference	4 (4.4)

^a^ At the time of the data collection, PCV13 was still in use. ^b^ At the time of the data collection, the age-based pneumococcal recommendation was for adults aged 65 or older.

**Table 3:** Frequency and prescription requirements for pneumococcal immunizations for specific age groups.

Patient Age Group	Frequency of Pharmacy-Administered Pneumococcal Vaccine; *n* (%)				Whether Prescription Is Required
	Never	Rarely	Sometimes	Often	
≤5 years old	89 (97.8)	2 (2.2)	-	-	2 (100)
5–18	80 (87.9)	8 (8.8)	3 (3.3)	-	8 (72.7)
19–64	7 (7.7)	24 (26.4)	49 (53.8)	11 (12.1)	23 (27.4)
≥65	-	3 (3.3)	13 (14.3)	75 (82.4)	5 (5.5)

**Table 4:** Methods used to identify patients and confirm eligibility (N = 91).

	*n* (%)
Strategies	
Vaccinate patients with pneumococcal vaccine when requested	90 (98.9)
Vaccinate patients with a prescription from their provider	75 (82.4)
Routinely assess vaccination status for patients who come in for other vaccines	69 (75.8)
Routinely assess the pneumococcal vaccination status	40 (44.0)
Proactively contact patients to schedule a pneumococcal vaccine	16 (17.6)
Data sources used to proactively identify eligible patients	
Patient age	68 (74.7)
Immunization Information Systems	65 (71.4)
Medication refill history	36 (39.6)
Data sources used to proactively confirm eligibility of patients	
Immunization Information Systems	83 (91.2)
Patient age	79 (86.8)
Medication refill history	56 (61.5)
Other ^a^	4 (4.4)
Resource used to help determine patient eligibility	
CDC immunization schedule	73 (80.2)
PneumoRecs Vax Advisor Website or Mobile App	34 (37.4)
Information from a previous training	31 (34.1)
Other ^b^	4 (4.4)

^a^ Patient self-report and insurance eligibility. ^b^ Immunization Information Systems.

**Table 5:** Patient-related and organizational barriers to pneumococcal immunization services (N = 91).

	*n* (%) ^a^
Patient-related barriers	
Patients refusing vaccine because they do not think they need it	3.1 (1.1)
Patients being too busy to discuss the vaccine with the pharmacist	2.7 (1.1)
Patient’s insurance coverage	2.6 (1.3)
Patients refusing the vaccine because they are concerned about immediate adverse reactions	2.6 (1.0)
Patients refusing because they do not trust vaccines	2.6 (1.2)
Patients refusing the vaccine because they feel they are not susceptible to pneumococcal disease	2.4 (1.1)
Patients refusing the vaccine because they are concerned about its long-term safety	2.2 (1.0)
Patient preferring to go to a non-pharmacy setting, such as medical clinic, for the vaccine	2.1 (1.0)
Patients refusing the vaccine because they feel pneumococcal disease is not a severe illness	2.1 (1.1)
Patients refusing because they have not heard about the vaccine	2.1 (1.0)
Patients refusing the vaccine because they are concerned about its efficacy	2.0 (1.0)
Organizational barriers	
Inadequate reimbursement for vaccine products	3.1 (1.5)
Inadequate reimbursement for vaccine administration	3.0 (1.5)
Cost of stocking the vaccine	2.5 (1.4)
Lack of a system to routinely screen patients for needed vaccines	2.3 (1.2)
Lack of time to counsel about the vaccine	2.0 (1.1)
Lack of a system to track multiple doses	2.0 (1.0)
Difficult to determine patient eligibility	1.9 (1.0)
Lack of support from other pharmacy personnel for immunization services	1.6 (1.0)
Lack of time to administer the vaccine	1.6 (0.9)
Lack of time to process vaccine reimbursements	1.6 (0.9)
Lack of staff certified to administer the vaccine	1.6 (0.9)
Lack of time to prepare the vaccine for administration	1.5 (0.8)
Difficult to integrate vaccine procedures into dispensing workflow	1.5 (0.9)
Vaccine is not usually in stock when needed	1.5 (0.9)
Difficult to access state immunization registry	1.4 (0.9)
Pharmacy unprepared to manage an adverse vaccine reaction	1.3 (0.7)

^a^ Response categories ranging from 1 to 5 (1 = not at all a barrier; 5 = an extreme barrier).

## Data Availability

The raw data supporting the conclusions of this article will be made available by the authors upon request.

## Supplementary Materials

The following supporting information can be downloaded at https://www.mdpi.com/article/10.3390/vaccines13070756/s1.

## Abbreviations

The following abbreviations are used in this manuscript:

US	United States
ACIP	Advisory Committee on Immunization Practices
RURAL-CP	Rural Research Alliance of Community Pharmacies
RUCA	Rural–Urban Commuting Area
PharmD	Doctor of Pharmacy
PCV13	Pneumococcal conjugate vaccine 13-valent
PCV15	Pneumococcal conjugate vaccine 15-valent
PCV20	Pneumococcal conjugate vaccine 20-valent
PCV21	Pneumococcal conjugate vaccine 21-valent
PPSV23	Pneumococcal polysaccharide vaccine 23-valent
CDC	Centers for Disease Control and Prevention
