# Supplementary material for: A Cross-Sectional Survey to Identify Current Pneumococcal Vaccination Practices and Barriers in Rural Community Pharmacies
Source: Vaccines (Basel). 2025 Jul 16;13(7):756. doi: 10.3390/vaccines13070756 (PMC12298712; doi:10.3390/vaccines13070756)
Supplement: Supplementary file 1 [file vaccines-13-00756-s001.zip › vaccines-3720197-supplementary.pdf]

Pneumococcal Vaccine Survey

Q1 Does your pharmacy currently administer any of the pneumococcal vaccines?

- No, and we do not plan to (1)
- Not currently, but we plan to (2)
- Yes (3)

Skip To: Q3 If Q1 = Yes

Page Break

Q2 Please elaborate on why your pharmacy does not currently administer pneumococcal vaccines:

____

____

____

____

____

Skip To: QID38 If Condition: Please elaborate on why you... Is Displayed. Skip To: The next set of questions asks about ....

Page Break

Q3 Which pneumococcal vaccines are you currently offering? *(Select all that apply).*

- ☐ PCV15 (1)
- ☐ PCV20 (2)
- ☐ PPSV23 (3)

Page Break

Q4 How many doses of the following vaccines did your pharmacy administer during the last calendar year (2023)? *Your best estimate is fine.*

- ○ PCV13: (1) ____
- ○ PCV15: (2) ____
- ○ PCV20: (3) ____
- ○ PPSV23: (4) ____

Page Break

Q5 How knowledgeable do you feel about the following vaccines?

	Not at all knowledgeable (1) (1)	Slightly knowledgeable (2) (2)	Somewhat knowledgeable (3) (3)	Moderately knowledgeable (4) (4)	Very knowledgeable (5) (5)
PCV15 (1)	○	○	○	○	○
PCV20 (2)	○	○	○	○	○
PPSV23 (3)	○	○	○	○	○

Q6 Have you heard of PCV21?

○ No (1)

○ Yes (2)

Page Break

Q7 Does your pharmacy co-administer pneumococcal vaccines with other vaccines?

○ No (1)

○ Yes (2)

Page Break

Q8 How often does your pharmacy administer pneumococcal vaccines to each of the following age groups?

	Never (0) (1)	Rarely (1) (2)	Sometimes (2) (3)	Often (3) (4)
Younger than 5 years old (1)	○	○	○	○
Ages 5-18 (2)	○	○	○	○
Ages 19-64 (3)	○	○	○	○
Age 65 and older (4)	○	○	○	○

Page Break

Q9 Does your pharmacy require a prescription before you administer a pneumococcal vaccine for the following age groups?

	No (1)	Yes (2)
Younger than 5 years old (1)	○	○
Ages 5-18 (2)	○	○
Ages 19-64 (3)	○	○
Age 65 and older (4)	○	○

Page Break

Q10 Has the demand for pneumococcal vaccines increased over the past calendar year at your pharmacy?

○ No (1)

○ Yes (2)

Page Break

Q11 What is your preferred pneumococcal vaccine regimen for adult patients aged 19 to 64 with certain high-risk conditions (e.g. asthma, diabetes, and heart disease) who have not previously received a pneumococcal vaccine? *(Select all that apply).*

○ PCV20 (1)

○ PCV15, followed by PPSV23 (2)

○ Other (please specify) (3) ____

○ No preference (4)

Page Break

Q12 What is your preferred pneumococcal vaccine regimen for adult patients aged 65 or older who have not previously received a pneumococcal vaccine? *(Select all that apply).*

○ PCV20 (1)

○ PCV15, followed by PPSV23 (2)

○ Other (please specify) (3) ____

○ No preference (4)

Page Break

The following section asks about patient communication and eligibility for pneumococcal vaccines.

Page Break

Q13 Which of the following does your pharmacy personnel do regarding pneumococcal vaccine administration? *(Select all that apply).*

▢ Proactively contact patients to schedule a pneumococcal vaccine (1)

▢ Routinely assess the pneumococcal vaccination status of patients who may be eligible for a pneumococcal vaccine (2)

▢ Routinely assess the vaccination status of patients who come in for other vaccines (3)

▢ Vaccinate patients with pneumococcal vaccine when they request the vaccine (4)

▢ Vaccinate patients with a prescription from their provider (5)

Page Break

Q14 Sometimes patients are not aware they should be getting a pneumococcal vaccine. Please indicate whether your pharmacy uses the following data sources to proactively identify patients who may be qualified to receive a pneumococcal vaccine *(Select all that apply).*

- ☐ Medication refill history (1)
- ☐ Patient age (2)
- ☐ Immunization registry (also known as Immunization Information System) (3)
- ☐ Other (please specify) (4) ____
- ☐ We do not proactively assess immunization status (5)

Page Break

Q15 Please indicate whether your pharmacy utilizes the following data sources to confirm patient eligibility to receive a pneumococcal vaccine *(Select all that apply)*.

- ☐ Medication refill history (1)
- ☐ Patient age (2)
- ☐ Immunization registry (also known as Immunization Information System) (3)
- ☐ Other (please specify) (4) ____

Page Break

Q16 What resource(s) do you use to help determine whether your adult patients are eligible to receive a pneumococcal vaccine? *(Select all that apply).*

- ☐ CDC immunization schedule (1)
- ☐ PneumoRecs VaxAdvisor Website or MobileApp (2)
- ☐ Information from a previous training (3)
- ☐ Other (please specify) (4) ____

Page Break

The following set of questions asks about factors that could influence pneumococcal vaccine administration at your pharmacy.

Page Break

Q17 How many of your pharmacy personnel, including yourself, are authorized to administer pneumococcal vaccines?

- ○ 1 (1)
- ○ 2 (2)
- ○ 3 (3)
- ○ More than 3 (4)

Page Break

Q18 Which of the following types of pharmacy personnel administered pneumococcal vaccines at your pharmacy in the calendar year 2023? *(Select all that apply).*

- ☐ Pharmacists (1)
- ☐ Pharmacy students on payroll (2)
- ☐ Pharmacy students on experiential rotations or other school experiences (3)
- ☐ Pharmacy technicians (4)
- ☐ Other (please specify) (5) ____

Page Break

Q19 Assuming adequate supply, what is the maximum number of pneumococcal vaccine doses your pharmacy would be capable of administering on a typical day? *Your best estimate is fine.*

- ○ 0 (1)
- ○ 1-10 (2)
- ○ 11-20 (3)
- ○ 21-30 (4)
- ○ More than 30 (5)

Page Break

Q20 Which of the following types of vaccines have been administered in your pharmacy in the last calendar year (2023)? *(Select all that apply).*

- ☐ Influenza (1)
- ☐ COVID-19 (2)
- ☐ Shingrix® (3)
- ☐ RSV (4)
- ☐ Other ACIP-recommended vaccines (5)

Page Break

The following section asks about barriers to providing pneumococcal vaccines in your pharmacy. *Please answer questions based on your local knowledge of your community.*

Page Break

Q21a Please indicate how much each of the following ***patient-related factors*** could be potential barriers to providing pneumococcal vaccines in your pharmacy.

	Not at all a barrier (1) (1)	A slight barrier (2) (2)	Somewhat of a barrier (3) (3)	A moderate barrier (4) (4)	An extreme barrier (5) (5)
Patients refusing vaccine because they do not think they need it (1)	○	○	○	○	○
Patient preferring to go to a non-pharmacy setting, such as medical clinic, for the vaccine (2)	○	○	○	○	○
Patients refusing the vaccine because they feel they are not susceptible to pneumococcal disease (3)	○	○	○	○	○
Patients refusing the vaccine because they feel pneumococcal disease is not a severe illness (4)	○	○	○	○	○
Patients being too busy to discuss the vaccine with the pharmacist (5)	○	○	○	○	○
Patient's insurance coverage (6)	○	○	○	○	○

Q21b Please indicate how much each of the following ***patient-related factors*** could be potential barriers to providing pneumococcal vaccines in your pharmacy.

	Not at all a barrier (1) (1)	A slight barrier (2) (2)	Somewhat of a barrier (3) (3)	A moderate barrier (4) (4)	An extreme barrier (5) (5)
Patients refusing the vaccine because they are concerned about immediate adverse reactions (1)	○	○	○	○	○
Patients refusing the vaccine because they are concerned about its long-term safety (2)	○	○	○	○	○
Patients refusing the vaccine because they are concerned about its efficacy (3)	○	○	○	○	○
Patients refusing because they have not heard about the vaccine (4)	○	○	○	○	○
Patients refusing because they do not trust vaccines (5)	○	○	○	○	○

Q22 What specific concerns or fears have patients voiced to you about pneumococcal vaccines? This could include things like not trusting any vaccine.

____

____

____

____

____

Page Break

Q23a Please indicate how much each of the following ***organizational/environmental factors*** could be potential barriers to providing pneumococcal vaccines in your pharmacy

	Not applicable (0) (1)	Not at all a barrier (1) (2)	A slight barrier (2) (3)	Somewhat of a barrier (3) (4)	A moderate barrier (4) (5)	An extreme barrier (5) (6)
Lack of support from other pharmacy personnel for immunization services (1)	○	○	○	○	○	○
Lack of time to counsel about the vaccine (2)	○	○	○	○	○	○
Lack of time to prepare the vaccine for administration (3)	○	○	○	○	○	○
Lack of time to administer the vaccine (4)	○	○	○	○	○	○
Lack of time to process vaccine reimbursements (5)	○	○	○	○	○	○
Pharmacy unprepared to manage an adverse vaccine reaction (6)	○	○	○	○	○	○

Page Break

Q23b Please indicate how much each of the following ***organizational/environmental factors*** could be potential barriers to providing pneumococcal vaccines in your pharmacy

	Not applicable (0) (1)	Not at all a barrier (1) (2)	A slight barrier (2) (3)	Somewhat of a barrier (3) (4)	A moderate barrier (4) (5)	An extreme barrier (5) (6)
Inadequate reimbursement for **vaccine administration (incentive fee)** (1)	o	o	o	o	o	o
Inadequate reimbursement for **vaccine product** (7)	o	o	o	o	o	o
Lack of physical space or a private area for vaccine administration (3)	o	o	o	o	o	o
Difficult to integrate vaccine procedures into dispensing workflow (4)	o	o	o	o	o	o
Lack of staff certified to administer the vaccine (5)	o	o	o	o	o	o
Difficult to access state immunization registry (6)	o	o	o	o	o	o

Page Break

Q23c Please indicate how much each of the following ***organizational/environmental factors*** could be potential barriers to providing pneumococcal vaccines in your pharmacy

	Not applicable (0) (1)	Not at all a barrier (1) (2)	A slight barrier (2) (3)	Somewhat of a barrier (3) (4)	A moderate barrier (4) (5)	An extreme barrier (5) (6)
Vaccine is not usually in stock when needed (1)	○	○	○	○	○	○
Cost of stocking the vaccine (3)	○	○	○	○	○	○
Difficult to determine patient eligibility (4)	○	○	○	○	○	○
Lack of a system to routinely screen patients for needed vaccines (5)	○	○	○	○	○	○
Lack of a system to track multiple doses (6)	○	○	○	○	○	○

Page Break

Q24 Is there anything else you think we should know regarding your ability to offer pneumococcal vaccines in your pharmacy?

____

____

____

____

____

Page Break

Q25 What, if anything, does your pharmacy need to better support your pneumococcal vaccination efforts?

____

____

____

____

____

Page Break

This next set of questions asks about the sources you use for pneumococcal vaccination information.

Page Break

Q26 Which sources do you use **the most** for pneumococcal vaccination information?

- ○ The Centers for Disease Control and Prevention (CDC) (1)
- ○ Pharmaceutical company website (2)
- ○ National or State Pharmacy/Pharmacists Association (3)
- ○ College or School of Pharmacy (4)
- ○ PneumoRecs VaxAdvisor (5)
- ○ Other source (please specify) (6) ____

Page Break

These questions ask you to compare vaccination barriers for pneumococcal vaccinations and shingles (Shingrix).

Page Break

Q27 Please indicate whether you think each of these factors is more of a barrier for pneumococcal vaccination **when compared to Shingrix vaccination**.

	This factor is less of a barrier for pneumococcal vaccination than for Shingrix (1) (1)	This factor is the same amount of a barrier for pneumococcal vaccination as for Shingrix (2) (2)	This factor is more of a barrier for pneumococcal vaccination than for Shingrix (3) (3)
Patients refusing vaccine because they do not think they need it (1)	○	○	○
Patients refusing the vaccine because they feel they are not susceptible to the illness the vaccine is trying to prevent (2)	○	○	○
Patients refusing the vaccine because they feel the illness the vaccine is trying to prevent is not a severe illness (3)	○	○	○
Patients refusing the vaccine because they are concerned about immediate adverse reactions (4)	○	○	○
Patients refusing the vaccine because they are concerned about its long-term safety (5)	○	○	○
Patients refusing the vaccine because they are concerned about its efficacy (6)	○	○	○
Patients refusing because they have not heard about the vaccine (7)	○	○	○

Q28a Please indicate whether you think each of these factors is more of a barrier for pneumococcal vaccination **when compared to Shingrix vaccination**.

	This factor is less of a barrier for pneumococcal vaccination than for Shingrix (1) (1)	This factor is the same amount of a barrier for pneumococcal vaccination as for Shingrix (2) (2)	This factor is more of a barrier for pneumococcal vaccination than for Shingrix (3) (3)
Lack of time to counsel about the vaccine (1)	○	○	○
Lack of time to prepare the vaccine for administration (2)	○	○	○
Lack of time to administer the vaccine (3)	○	○	○
Lack of time to process vaccine reimbursements (4)	○	○	○
Inadequate reimbursement for vaccine (5)	○	○	○
Inadequate reimbursement for vaccine administration (6)	○	○	○
Difficulty in integrating vaccine procedures into dispensing workflow (7)	○	○	○

Page Break

Q28b Please indicate whether you think each of these factors is more of a barrier for pneumococcal vaccination **when compared to Shingrix vaccination**.

	This factor is less of a barrier for pneumococcal vaccination than for Shingrix (1) (1)	This factor is the same amount of a barrier for pneumococcal vaccination as for Shingrix (2) (2)	This factor is more of a barrier for pneumococcal vaccination than for Shingrix (3) (3)
Lack of staff certified to administer the vaccine (1)	○	○	○
Vaccine is not usually in stock when needed (2)	○	○	○
Patient's insurance coverage (3)	○	○	○
Cost of stocking the vaccine (4)	○	○	○
Difficulty to determine patient eligibility (5)	○	○	○
Lack of a system to routinely screen patients for needed vaccines (6)	○	○	○
Lack of a system to track multiple doses (7)	○	○	○

Page Break

The next set of questions asks about your pneumococcal vaccination training preferences.

Page Break

Q29 Which types of pneumococcal vaccination training have you had in the past 2 years in addition to the immunization certificate training course? *(Select all that apply).*

- ▢ Pneumococcal vaccine-specific CE (1)
- ▢ Annual immunization update CE (2)
- ▢ Reviewed the MMWR/ACIP Recommendations for pneumococcal vaccination (3)
- ▢ Other (please specify) (4) ____
- ▢ I have not had additional pneumococcal vaccination training (5)

Page Break

Q30 What training (if any) would you like to have about the pneumococcal vaccines? *(Select all that apply).*

- ☐ Identifying patient populations for the vaccine (1)
- ☐ Injection techniques (2)
- ☐ Reporting to the state immunization registry (3)
- ☐ Reporting adverse events to the Vaccine Adverse Event Reporting System (VAERS) (4)
- ☐ Reporting vaccine administration errors to the Institute for Safe Medication Practices National Vaccine Errors Reporting Program (ISMP VERP) (5)
- ☐ Communicating with patients about the vaccines (6)
- ☐ Shared clinical decision making for patients who previously completed a pneumococcal vaccine series (7)
- ☐ Addressing patients' vaccine hesitancy (8)
- ☐ Payment and billing (9)
- ☐ Liability coverage (10)
- ☐ Vaccine marketing techniques (11)
- ☐ Storage and handling of the vaccine (12)
- ☐ Preparing the vaccine for injection (13)
- ☐ Vaccine safety data (14)
- ☐ Mechanism of action (15)
- ☐ Other (please specify) (16) ____

☐ None (17)

Page Break

Q31 What would be the best way to deliver pneumococcal vaccine training? *(Select all that apply).*

☐ Webinar with live CE credit (1)

☐ Online course with correspondence CE credit (2)

☐ Written materials (3)

☐ Website with training and resources (4)

☐ Other (Please specify) (5) ____

☐ Not Interested in pneumococcal vaccine training (6)

Page Break

This next set of questions asks about your personal opinions about pneumococcal vaccines.

Page Break

Q32 Would you recommend a pneumococcal vaccine to the following types of patients?

	Yes (1)	No (2)	Not Sure (3)
People **younger than 19** at high risk for pneumococcal disease due to underlying medical conditions (1)	○	○	○
People **age 19 and older** at high risk for pneumococcal disease due to underlying medical conditions (2)	○	○	○
People 65 years and older (3)	○	○	○

Page Break

Q33 How confident are you that you could answer patient questions about getting a pneumococcal vaccine?

- ○ Not at all confident (1)
- ○ A little confident (2)
- ○ Moderately confident (3)
- ○ Very confident (4)

Page Break

Q34 How much will you want to get a pneumococcal vaccine when you are eligible to receive it?

- ○ Not at all (1)
- ○ A little (2)
- ○ Moderately (3)
- ○ Very much (4)

Page Break

Q35 Please tell us to what extent you agree or disagree with the following statements regarding the pneumococcal immunization schedule published by CDC.

	Strongly Agree (1)	Somewhat Agree (2)	Somewhat Disagree (3)	Strongly disagree (4)	Not familiar with this (5)
I am comfortable using the immunization schedule to determine whether my adult patients need a pneumococcal vaccine. (1)	○	○	○	○	○
I am comfortable using the immunization schedule to determine which pneumococcal vaccine my adult patients need. (2)	○	○	○	○	○
The age-based indications for the pneumococcal vaccine on the schedule are difficult to follow. (3)	○	○	○	○	○
The medical condition-based indications on the schedule are difficult to follow. (4)	○	○	○	○	○
The schedule is easily accessible when I need it to make a decision about a patient’s vaccinations. (5)	○	○	○	○	○
The schedule provides clear guidance on what to do when immunization status is unknown. (6)	○	○	○	○	○

The footnote section of the schedule is clear and concise. (7)	○	○	○	○	○
The schedule provides clear guidelines on catch-up vaccinations for adults. (8)	○	○	○	○	○
I do not use the schedule to guide my vaccine recommendations. (9)	○	○	○	○	○

Page Break

The last set of questions asks about demographic characteristics and characteristics of your pharmacy.

Page Break

Q36 How old are you?

____

Page Break

Q37 What is your gender?

- ○ Female (1)
- ○ Male (2)
- ○ Non-binary or transgender (3)
- ○ Prefer not to answer (4)

Page Break

Q38 Which of the following best describes your race/ethnicity? *(Select all that apply).*

- ☐ Asian or Pacific Islander (1)
- ☐ Black or African American (2)
- ☐ Hispanic or Latinx (3)
- ☐ Native American or Alaska Native (4)
- ☐ White or Caucasian (5)
- ☐ Multiracial or Biracial (6)
- ☐ Other (Please specify) (7) ____
- ☐ Prefer not to answer (8)

Page Break

Q39 What is your highest level of pharmacy education?

- ○ BSPharm (1)
- ○ MSPharm (2)
- ○ PharmD (3)
- ○ Other (Please specify) (4) ____

Page Break

Q40 How long have you worked in pharmacy practice as a pharmacist?

- ◯ Less than 1 year (1)
- ◯ 1-3 years (2)
- ◯ 4-7 years (3)
- ◯ 8-11 years (4)
- ◯ 12-15 years (5)
- ◯ Greater than 16 years (6)

Page Break

Q41 What is your role in the pharmacy?

- ◯ Pharmacy Manager (1)
- ◯ Pharmacist (2)
- ◯ Other (Please specify) (3) ____

Page Break

Q42 What type of pharmacy do you work at?

- ○ A single independent pharmacy (1)
- ○ An independent pharmacy with multiple locations (2)
- ○ Grocery store chain (3)
- ○ Regional chain (4)
- ○ National chain (5)
- ○ Other (Please specify) (6) ____

Page Break

Q43 What is the zip code at your pharmacy?

____

Page Break
